# Does tenodesis of tensor fascia latae with hip abductors after proximal femoral resection and modular endoprosthetic reconstruction lead to functional improvements?

**DOI:** 10.1002/jeo2.70534

**Published:** 2025-12-07

**Authors:** Ariane Lavoie‐Hudon, Jean‐Philippe Cloutier, Norbert Dion, Simon Laurendeau, Philippe Corbeil, Annie Arteau

**Affiliations:** ^1^ Groupe de Recherche en Analyse du Mouvement et Ergonomie (GRAME), Department of Kinesiology Université Laval Québec Canada; ^2^ Centre Interdisciplinaire de Recherche en Réadaptation et Intégration Sociale (CIRRIS) Québec Canada; ^3^ Division of Orthopaedic Surgery, Department of Surgery CHU de Québec‐Université Laval Quebec Canada

**Keywords:** electromyography (EMG), endoprosthesis, gait analysis, patient‐related outcome measures (PROMs), tensor fascia latae (TFL)

## Abstract

**Purpose:**

Trendelenburg gait is a common consequence of proximal femoral oncologic resection. To mitigate limping, tensor fascia latae (TFL) tenodesis is employed by suturing the muscle to the abductor mechanism, repaired to the trochanteric portion of the endoprosthesis. Objective functional evaluation of this technique has not been conducted. This study aimed to determine (1) whether the procedure induces tensor fascia latae hypertrophy, (2) its impact on functional outcomes and (3) its effect on gait patterns.

**Methods:**

Sixteen patients who underwent proximal femoral resection and modular endoprosthesis reconstruction were assessed via computed tomography scans at least 1 year post‐operatively for TFL and hip abductor hypertrophy and fatty infiltration. Patients were separated into two groups based on the presence or absence of TFL hypertrophy. Patient‐related outcomes were evaluated with questionnaires, and a subset of seven patients underwent hip abductor strength measurement and gait analysis to assess objective function. Gait analysis included kinematics as well as electromyography.

**Results:**

At 1 year, half of the cohort demonstrated TFL hypertrophy. A trend towards improved functional scores was observed in the hypertrophy group. Hip kinematics indicated a greater adduction (max of 7.2 ± 4.1° vs. 2.8 ± 2.6°, 88% difference) in the hypertrophy group, resulting in an increased pelvic drop during single‐limb support (5.2 ± 3.1° in the hypertrophy group and 3.4 ± 3.7° in the no hypertrophy group, 42% difference). Gluteus medius activation tended to be slightly greater during the stance phase for the no hypertrophy group, while the TFL was most activated in the hypertrophy group in the same period.

**Conclusion:**

The TFL tenodesis led to satisfactory functional outcomes for patients with proximal femoral reconstruction, whether they developed hypertrophy or not. TFL hypertrophy was not associated with a more favourable gait pattern, despite positive self‐evaluated function.

**Level of Evidence:**

Level IV.

**Abbreviations**
APC: allograft‐prosthetic composite
CSA: cross‐sectional area
EMG: electromyography
FI: fatty infiltration
GMax: gluteus maximus
GMed: gluteus medius
GMin: gluteus minimus
H+/H−: hypertrophy‐positive or negative
MEP: modular endoprosthesis
TFL: tensor fascia latae

## INTRODUCTION

### Background

Hip reconstruction in complex or revision total hip arthroplasty (THA) requires attention to both implant stability and soft‐tissue function. Several studies highlight the multifactorial nature of hip reconstruction and the need for tailored approaches in patients undergoing surgery for musculoskeletal tumours. Patient‐related risk factors, such as frailty, anaemia and hypoalbuminemia, increase the likelihood of early dislocation following THA [28]. In the setting of large acetabular defects, Cimatti et al. reported favourable outcomes using morselized bone allografts, with low failure rates across various fixation methods [8]. In oncologic cases, Henderson et al. found that instability after proximal femoral endoprosthesis is influenced by patient age, diagnosis and surgical technique, with synthetic soft‐tissue augmentation offering improved stability [21].

The proximal femur is a common site for primary and metastatic malignant bone tumours. Surgical treatment may require extensive resection of the proximal femur for oncological or mechanical reasons [4]. The loss of abductor mechanism insertion following proximal femur resection can result in instability of the hip joint, Trendelenburg gait and impaired patient function [21]. As survival of patients with musculoskeletal tumours has improved over the years, investigating techniques which improve patients' functional outcomes has become essential.

The two most common bone reconstruction techniques of the proximal femur are modular endoprosthesis (MEP) and allograft‐prosthetic composite (APC) reconstruction [4, 7, 13, 35]. Abductor mechanism reconstruction following endoprosthetic reconstruction is still a matter of debate. Four different types of abductor muscle reconstruction have been described: direct muscle repair to the prosthesis, trochanteric osteotomy, muscle‐to‐muscle suture, and synthetic tube reconstruction around the prosthesis [23]. Osteotomy and preservation of greater trochanter with direct fixation to the prosthesis was traditionally considered the optimal reconstruction technique, but recently, it has been shown that it does not improve the Trendelenburg gait or reliance on an assistive ambulatory device [19, 30, 32]. Conversely, indirect reattachment of muscle to a mesh or tube sutured around the prosthesis may decrease Trendelenburg gait and improve function [30], while direct fixation of abductor muscles to the prosthesis improved functional outcome if compared to abductor muscles reinsertion to the fascia latae only [16]. Direct muscle reattachment to the prosthesis, combined with muscle‐to‐muscle reconstruction, can also be performed, the objective being to generate power and motion from every abductor muscle around the joint.

Hip abduction is normally carried out by the gluteus medius (GMed) and minimus (GMin) working synergistically, especially to achieve stability during single‐limb support [17]. The tensor fascia latae (TFL), as a third abductor, mainly performs active abduction [17], suggesting that the TFL may assume a compensatory function in patients with compromised hip abduction. Furthermore, hypertrophy of the TFL following abductor tendon tears has been observed in some patients [34]. Recently, gait analysis has emerged as an evaluation instrument for the functional outcome of limb salvage procedures. Only a few studies focused on patients with hip replacement for bone tumour resections [3, 11, 12, 24, 33]. Benedetti et al. compared function and gait analysis between APCs and MEPs [3] with the GMed directly reattached to the prosthesis, but no testing was reported on TFL or abductor muscles. Trendelenburg gait has often been used to gauge hip abductor weakness, but there is poor agreement between clinicians' evaluations and objective three‐dimensional (3D) measures of the pelvis [29]. Gait analysis and direct strength assessment may provide more accurate, objective measures of the abductor mechanism's function.

### Rationale

The present study was conducted to determine (1) whether the TFL tenodesis to abductor muscles in patients with modular endoprosthetic reconstruction following proximal femoral resection can lead to TFL hypertrophy; (2) whether this hypertrophy carries objective and patient‐reported functional benefits and (3) whether TFL hypertrophy in these patients results in an improved gait pattern. The hypothesis was that TFL tenodesis leads to hypertrophy of the TFL in some patients, and that these patients have better functional outcomes than patients who do not develop hypertrophy. To our knowledge, it is the first time TFL muscle hypertrophy and gait analysis are evaluated on patients following an oncologic resection of the proximal femur.

## MATERIALS AND METHODS

### Study population and design

**Figure 1:**
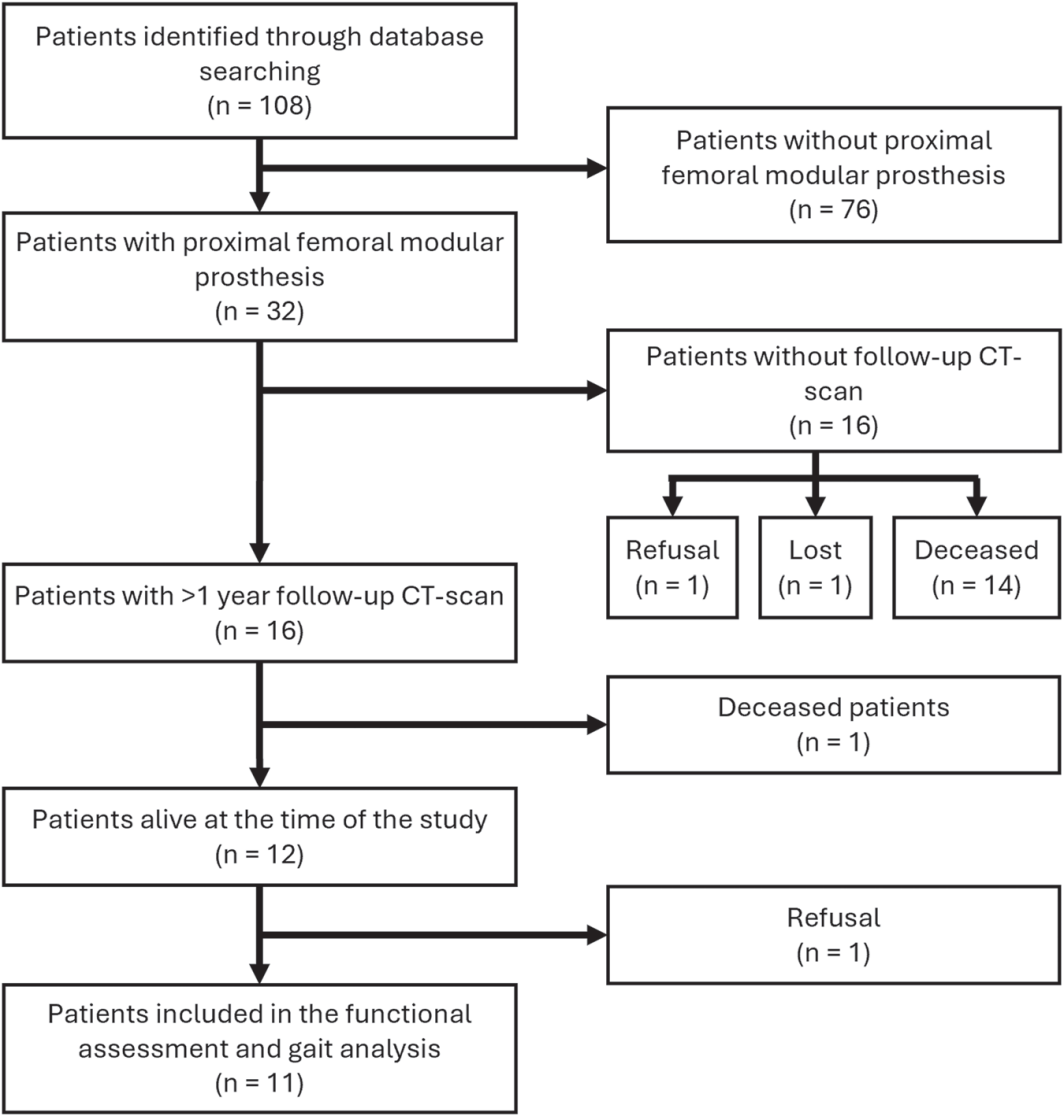
Flowchart of patients' inclusion. CT, computed tomography.

Demographic and surgical data were retrospectively collected from patients who sustained proximal femoral resection and endoprosthetic reconstruction between 2001 and 2016 at the same institution by two fellowship‐trained oncologic orthopaedic surgeons (ND and AA). During this period, the TFL tenodesis technique described below was the standard protocol for all patients. Patients were identified through the local database. The inclusion criteria were: 18 years of age or older at the time of surgery, unilateral proximal femoral resection required for cancer, Stryker's Global Modular Replacement System® (GMRS, Stryker) endoprosthesis reconstruction, and a minimal 1‐year follow‐up with pelvic computed tomography (CT) scans available for review (Figure 1). Patients who had bilateral involvement or surgery on the contralateral side were excluded. Some information was inconsistently reported in patients' medical files, including complications, tumour size and soft tissue involvement. Consequently, we confined our formal analysis to fully complete data, while other relevant information is reported as it appears in the files. All patients alive at least 1 year post‐operatively who were willing to participate in the functional assessment were included.

### Ethics approval

All procedures in this project were performed in compliance with relevant laws and institutional guidelines and have been approved by the institutional ethics committee. All participants provided their written informed consent prior to participating, and their rights to privacy have been observed.

### Surgical technique

**Figure 2:**
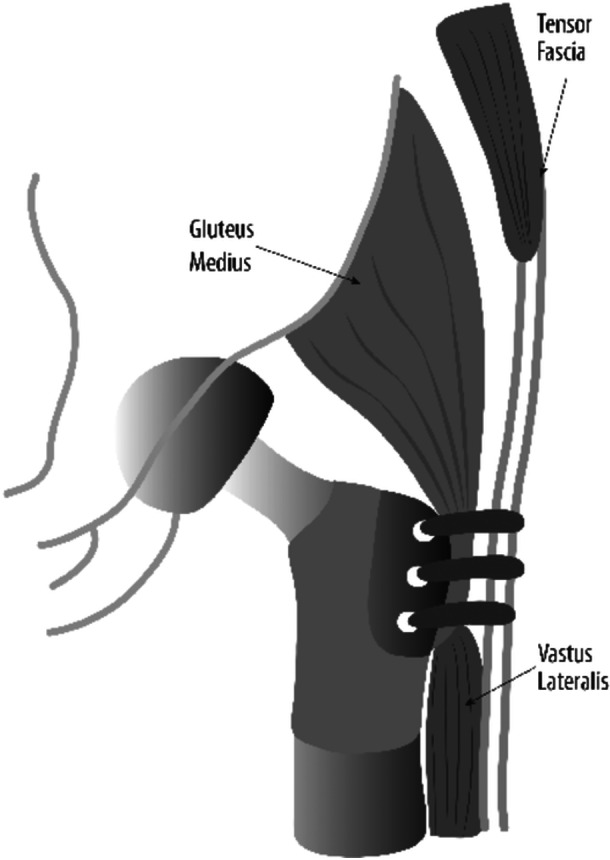
Illustration of the surgical technique.

Patients in this study underwent an intra‐articular resection of the proximal femur. The rectus femoris was always spared, and the iliopsoas was detached from the proximal femur and left free. If it were oncologically safe, the GMed and GMin were detached from the greater trochanter in continuity with the vastus lateralis by subperiosteal dissection to avoid proximalization of the abductor muscles. After resection, the abductor tendons were sutured to the prosthesis with Mersilene tapes (©Ethicon US, LLC). The iliotibial band was also sutured to the gluteus medius tendon as well as to the trochanteric portion of the prosthesis, forming a single unit (Figure 2). The reorientation of the tensor fascia lata to the prosthesis formed a tenodesis between the abductor muscles and the TFL. Importantly, this is achieved without mobilisation of the TFL muscle belly itself; in normal anatomy, the iliotibial band and TFL insert solely on Gerdy's tubercle, but by adding a robust fixation on the prosthesis and tenodesing the band to the gluteus medius, a second point of insertion is created. This effectively shortens the TFL's lever arm and allows it to function synergistically with the gluteus medius to counteract pelvic drop. A similar technique was described for reattachment of the abductor tendon onto the allogenic tendon of APC constructs [35].

By tensioning the TFL as a secondary anchor, the technique is intended to lessen the load on the gluteus medius and support frontal plane stability. Moreover, with leg length anatomically restored using the modular prosthesis, there is no undue tension on the repaired gluteus medius or TFL; anchoring at the trochanteric level restores proper muscle length and tension, maximizing power generation around the joint.

There are no contraindications to this reconstruction except in patients with extensive gluteus or fascia lata resection, which render the technique unfeasible. Otherwise, it can be readily combined with other abductor‐reconstruction methods, such as allograft–prosthesis composites or mesh supplementation.

### Radiographic assessments

**Figure 3:**
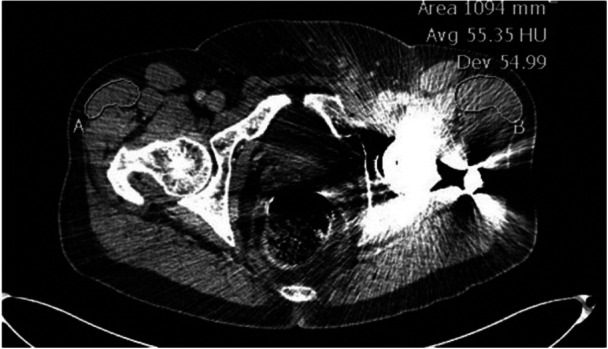
Example of the outlined TFL muscles in patient with TFL hypertrophy. Zone A shows the unoperated TFL outline, and Zone B shows the hypertrophied TFL outline on the surgical side. TFL, tensor fascia latae.

Radiographic measures were made on post‐operative pelvic CT scans obtained at least 1 year after surgery. The level of each CT‐scan slice was selected as a fixed percentage (20%, 40%, 60% and 80%) of the distance between the highest point on the iliac crest and the lowest level on the ischium based on a previously described method [9]. Muscle volume and fatty infiltration (FI) of the TFL, GMin, GMed, gluteus maximus (GMax) and iliopsoas were estimated to describe muscle quality. Axial cross‐sectional area (CSA) of each muscle was measured on the four standardized CT‐scan slices. Bilateral cross‐sectional areas were manually outlined using the Impax 6 software (©2017 Agfa‐Gevaert Group) by two independent observers (JPC and LOB) (Figure 3), then measured in square millimetres. For each slice, the mean value between observers was computed. Hypertrophy of the TFL was defined as a 10% increase in CSA on any of the four slices. Only the peak muscle CSA among the slices were reported and used for comparison; it was not necessarily the same slice for each muscle. Muscle atrophy was calculated as follows:

$$\%\mathrm{Atrophy}=\frac{{\mathrm{Healthy}}_{\mathrm{CSA}}-{\mathrm{Operated}}_{\mathrm{CSA}}}{{\mathrm{Healthy}}_{\mathrm{CSA}}}\times 100$$

FI of the muscles of interest was then measured using mean pixel density (Hounsfield unit [HU]). Mean pixel density is a measure of radiation attenuation on CT scans. Because of the difference in the radiation attenuation range of adipose tissue (−190 to −30 HU) and muscle (−30 to 150 HU), the mean pixel density decreases as FI in the muscle increases [1]. This measure was averaged over all slices available for each muscle.

### Functional assessments

Of the 16 patients, 11 were still alive at the time of the study and were available for gait analysis. Seven gait trials were recorded at a self‐selected comfortable walking speed for each participant. Measurements were taken by 14 infrared cameras (FLEX: V100R2, NaturalPoint Inc.). Forty‐two markers were fixed on anatomical landmarks of the lower limbs (iliac crest, anterosuperior iliac spines, greater trochanters, lateral and medial femoral epicondyles, lateral and medial malleoli, heels and fifth metatarsal joints) [6]. Kinematic data were collected at a frequency of 100 Hz. Surface electromyographic (EMG) data were collected with electrodes applied bilaterally over TFL and GMed to evaluate muscle activation during walking cycles [26, 27]. The positioning of the electrodes followed SENIAM recommendations [22].

Processing of kinematic and EMG data was conducted in Visual3D (HAS‐Motion, ON, Canada). A fourth‐order, zero‐phase shift, low‐pass Butterworth filter with a cut‐off frequency of 8 Hz was applied to the 3D motion data before joint angle calculation. This data was used to create a 7‐segment model of the lower body in Visual 3D. Cardan angles equivalent to the Joint Coordinate System proposed by Grood and Suntay [18] were used to compute joint angles. Gait parameters, such as walking velocity, step length and stance phase duration, were extracted from the trials in Visual 3D. Pelvic angles (obliquity, rotation and tilt) were computed by creating landmarks to generate angles between the anterior superior iliac spines and the horizontal plane of the lab in the three planes [2]. EMG activity of each muscle was rectified, smoothed with a 6 Hz low‐pass Butterworth filter and normalized with the peak activity during the stance phase. All variables were then normalized to 100% of the gait cycle. The mean of the seven trials was computed for each condition for each participant prior to analysis.

The quantitative evaluation of the muscle strength in abduction was measured for 3 s using a Lafayette dynamometer (©Lafayette Instrument) apposed on the lateral femoral condyle and perpendicular to the femur axis with the patient in supine position based on the technique described by Giurea et al. [16, 25]. The abductor muscle strength was measured three times, and a mean value in kilograms was calculated. Operated and non‐operated limbs were tested by a qualified physical therapist.

The clinical function of the patients was also assessed using the Harris Hip score, the Musculoskeletal Tumor Society (MSTS) scoring system, the Ambulation score and the Toronto extremity salvage score (TESS) [5, 10, 14, 20, 33].

### Statistical analysis

Patients' and surgery factors as well as functional scores from questionnaires were compared between groups: for continuous variables, an unpaired *t* test was applied; for categorical variables, a Fisher's exact test was used. Intraclass correlation was computed for radiographic measures of FI and muscle volume. Muscle CSA, FI and abductors strength were tested for normality with the Lilliefors' test and log‐corrected when necessary. If log transformation failed to correct the distribution, non‐parametric tests were used for the comparison of dependent variables (sides) and/or independent variables (groups): the Wilcoxon test and Mann–Whitney *U* test, respectively. Otherwise, the Levene's test was applied to the normally distributed data to evaluate the homogeneity of variance between groups. If no difference in variance was found, a mixed ANOVA was applied to compare means between the groups, with the side (operated or healthy limb) as a within‐factor. The level of significance was set at 0.05. Considering the reduced number of participants who underwent gait testing, comparisons between groups and within repeated measures were made using a descriptive analysis. The mean and standard deviation (SD) of their gait parameters, joint angular displacement, pelvic angles and muscle activity over a full gait cycle were computed. All data are reported as mean ± SD unless stated otherwise. Statistical analyses were conducted in RStudio (Posit Software).

## RESULTS

### TFL tenodesis to abductor muscles and TFL hypertrophy

In the 16 patients who had a proximal femoral resection and MEP reconstruction, 8 had hypertrophy of the TFL muscle on their operative side (Table 1). The group with TFL hypertrophy on the operative side (H+) consisted of three men and five women. The mean age at surgery was 60 years. The mean resection length was 14.3 (12.0) cm. Five patients had surgery for sarcoma and two for metastatic disease. The group without hypertrophy of the TFL (H‐) consisted of six men and two women. The mean age at surgery was 55 years. The mean length resection was 15.4 (14.5) cm. Four patients had surgery for sarcoma, two for metastatic disease and two for non‐union of a pathologic fracture through irradiated bone.

**Table 1:** Patients' and surgical factors.

		H+ (*n* = 8)	H− (*n* = 8)	*p*
Patients	Age at the time of the study (years [median])	66 ± 10 (62)	59 ± 14 (57)	0.27
	Age at index surgery	60 ± 10 (58)	55 ± 14 (55)	0.42
	Sex (*n* [%])			
	Male	3 (38)	6 (75)	0.31
	Female	5 (62)	2 (25)	
	Indication (*n* [%])			
	Sarcoma	5 (62)	4 (50)	0.60
	Metastasis	3 (38)	2 (25)	
	Non‐union	0 (0)	2 (25)	
	ASA (*n* [%])			
	1	1 (12)	1 (12)	0.08
	2	7 (88)	3 (38)	
	3	0 (0)	4 (50)	
Surgery	Resection length (cm [median])	14.3 ± 6 (12)	15.4 ± 6 (14.5)	0.72
	Muscle resection (*n* [%])	2 (25)	2 (25)	N/A
	Blood loss (mL [median])	631.3 ± 10 (625)	956.3 ± 1174 (600)	0.45

Abbreviation: ASA, American Society of Anesthesiologists.

Four patients underwent significant muscle resection in the quadriceps. No patients had substantial resection of the GMin, GMed, GMax or TFL. Among the 16 patients, complications included one pulmonary embolism, one case of delirium, and two requiring blood transfusions. Additionally, one patient underwent revision surgery 2 years later due to aseptic loosening. No complications were directly associated with the tenodesis technique, and no instances of stent dislocation or infection were observed.

The intraclass correlation was between 0.84 and 0.97 for all the radiographic measures. Muscle FI was similar between the two groups (Table 2). For both H+ and H− patients, the GMin, GMed and iliopsoas had more FI on the operated side (*p* < 0.05); in contrast, the TFL had less FI on the operated side in both groups (*p* < 0.001).

**Table 2:** Fatty infiltration (FI) of each muscle on the operated and healthy sides of patients in the hypertrophy (H+) and no hypertrophy (H−) groups.

	H+ (*n* = 8)		H− (*n* = 8)		*p*		
	Healthy	Operated	Healthy	Operated	Group	Side	Interaction
FI (HU)^a^							
TFL	16.9 ± 17.6	76.3 ± 44.7	26.2 ± 14.1	72.2 ± 43.9	0.96	**<0.001**	0.56
GMin	30.5 ± 28.4	−7.8 ± 23.9	34.5 ± 12.6	7.6 ± 26.8	0.55	**<0.001**	0.64
GMed	26.1 ± 14.5	−15.4 ± 23.1	33.0 ± 16.1	3.9 ± 18.4	0.23	**<0.001**	0.27
GMax	2.9 ± 18.4	13.7 ± 31.4	17.4 ± 11.2	20.8 ± 18.1	0.40	0.13	0.42
Iliopsoas	47.6 ± 14.5	27.2 ± 22.5	49.5 ± 9.7	42.6 ± 22.5	0.42	**0.04**	0.26

*Note*: Bold values denote statistical significance (*p* < 0.05).

Abbreviation: HU, Hounsfield unit.

a Smaller HU values denote greater FI in the muscle.

All patients showed atrophy of different magnitudes in every muscle surrounding the endoprosthetic reconstruction, except for the TFL (Table 3). A significant interaction showed that the TFL of the H+ group had an average hypertrophy of 24 ± 32%, while H− patients experienced an average atrophy of 17 ± 23% (*p* = 0.01). Every other muscle (GMin, GMed, GMax and iliopsoas) was atrophied by 14%–42% on the operated side compared to the healthy side for both groups. A significant interaction was found showing that the GMed and GMax CSA difference between sides was smaller in the H+ group, and that the CSA was smaller on the healthy side for the H+ group than on the healthy side for the H− group (*p* < 0.02).

**Table 3:** Cross‐sectional area (CSA) of each muscle on the operated and healthy sides of patients in the hypertrophy (H+) and no hypertrophy (H−) groups.

	H+ (*n* = 8)		H− (*n* = 8)		*p*		
	Healthy	Operated	Healthy	Operated	Group	Side	Interaction
CSA (mm^2^)							
TFL	507 ± 183	674 ± 395	605 ± 120	497 ± 143	0.59	0.61	**0.01**
GMin	893 ± 344	598 ± 206	1050 ± 221	704 ± 186	0.72	**<0.001**	0.84
GMed	2243 ± 637	1818 ± 572	3029 ± 630	2004 ± 545	0.12	**<0.001**	**0.02**
GMax	3308 ± 608	2856 ± 805	4261 ± 1026	3171 ± 901	0.22	**<0.001**	**0.01**
Iliopsoas	1252 ± 547	730 ± 377	1930 ± 390	1139 ± 373	0.09	**<0.001**	0.20

*Note*: Bold values denote statistical significance (*p* < 0.05).

Abbreviations: GMax, gluteus maximus; GMed, gluteus medius; GMin, gluteus minimus; TFL, tensor fascia latae.

### Muscle strength and patient‐reported outcomes

Most patients were weaker in abduction when measured with the dynamometer on their operative side compared to their non‐operative side, and these differences were significant (*p* = 0.005, Table 4). For the functional scores, a trend was noted towards better function in the H+ group. Harris hip score, TESS score, Ambulation score and MSTS score were higher in the H+ patients (Table 5).

**Table 4:** Mean abduction strength in the hypertrophy (H+) and no hypertrophy (H−) groups.

	H+ (*n* = 5)		H− (*n* = 6)		*p* a	
	Healthy	Operated	Healthy	Operated	Group	Side
Strength (kg)	17.5 ± 4.8	14.4 ± 5.2	20.6 ± 8.2	14.8 ± 7.0	0.39	**0.005**

*Note*: Bold values denote statistical significance (*p* < 0.05).

a Results of the non‐parametric tests.

**Table 5:** Mean functional scores in the hypertrophy (H+) and no hypertrophy (H−) groups.

	H+ (*n* = 5)	H− (*n* = 6)	*p*
Score (%)			
Harris	81 ± 14	70 ± 14	0.18
Ambulation 1	69 ± 9	56 ± 15	0.20
Ambulation 2	68 ± 11	52 ± 27	0.41
TESS	77 ± 15	74 ± 16	0.79
MSTS	84 ± 7	75 ± 10	0.12

Abbreviations: MSTS, Musculoskeletal Tumor Society; TESS, Toronto extremity salvage score.

### Gait pattern with TFL hypertrophy

Four patients were excluded from the final gait analysis because of their inability to walk without assistive device. In total, the walking pattern data from seven patients were analyzed: three H+ patients and four H− patients. Patients without hypertrophy tended to walk with a larger stride width than patients with hypertrophy (Table 6). Step length, stance phase duration and swing time duration were not meaningfully different between the limbs or between the groups.

**Table 6:** Gait parameters in the hypertrophy (H+) and no hypertrophy (H−) groups.

	H+ (*n* = 3)		H− (*n* = 4)	
Gait speed (m/s)	0.91 ± 0.31		1.01 ± 0.32	
Stride width (m)	0.09 ± 0.02		0.13 ± 0.02	

	Healthy	Operated	Healthy	Operated
Step length (m)	0.60 ± 0.07	0.60 ± 0.05	0.63 ± 0.07	0.64 ± 0.09
Stance duration (s)	0.93 ± 0.36	0.89 ± 0.29	0.75 ± 0.11	0.74 ± 0.10
Swing duration (s)	0.48 ± 0.10	0.52 ± 0.18	0.42 ± 0.03	0.45 ± 0.06

**Figure 4:**
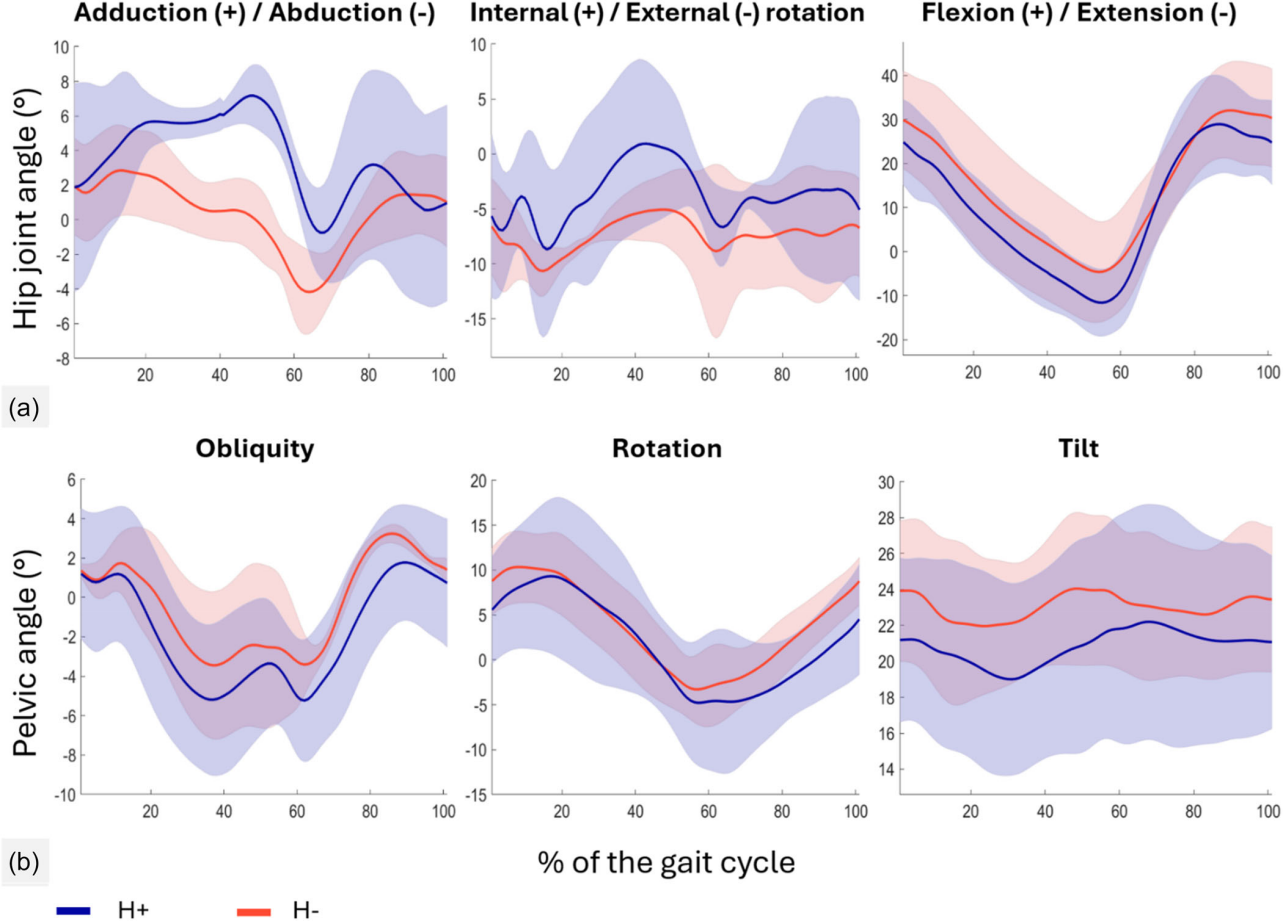
Kinematics of the operated hip and the pelvis over the gait cycle. Solid lines represent the mean joint angles of the operated hip (a) and the mean pelvic angle (b), for the TFL hypertrophy (H+) and no hypertrophy (H−) groups. The lighter coloured areas depict standard deviation. TFL, tensor fascia latae.

**Figure 5:**
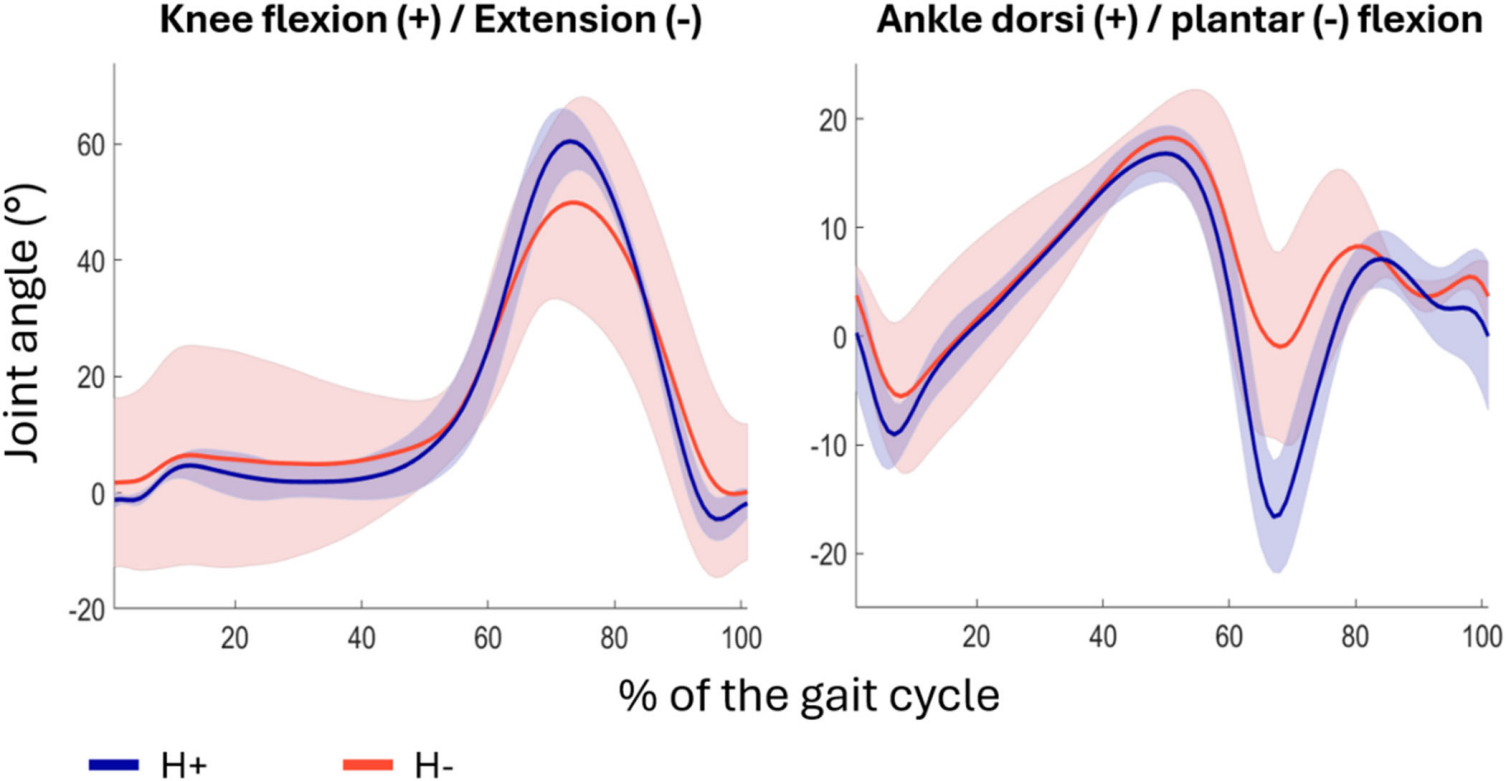
Kinematics of the operated knee and ankle over the gait cycle. Solid lines represent the mean joint angles of the knee and ankle for the TFL hypertrophy (H+) and no hypertrophy (H−) groups in the sagittal plane. The lighter coloured areas depict standard deviation. TFL, tensor fascia latae.

Hip kinematics indicated a greater adduction (max of 7.2 ± 4.1° vs. 2.8 ± 2.6°, 88% difference) and slightly greater internal rotation of the femur in the H+ group, especially during single‐limb support (Figure 4a). Pelvic drop during single limb support, as measured by pelvic obliquity, reached 5.2 ± 3.1° in the H+ group and 3.4 ± 3.7° in the H− group (42% difference). The pelvic drop was also apparent for greater portion of the gait cycle for the H+ group compared to the H‐ group. Obliquity measure was lesser than 0° in the H+ group for an average of 63% of the gait cycle versus 50% in the H− group (Figure 4b). Both knee flexion and ankle plantarflexion were greater in the H+ group following push‐off (Figure 5). Kinematics of the healthy limb are available in Supporting Information (see Figure S1–S3).

**Figure 6:**
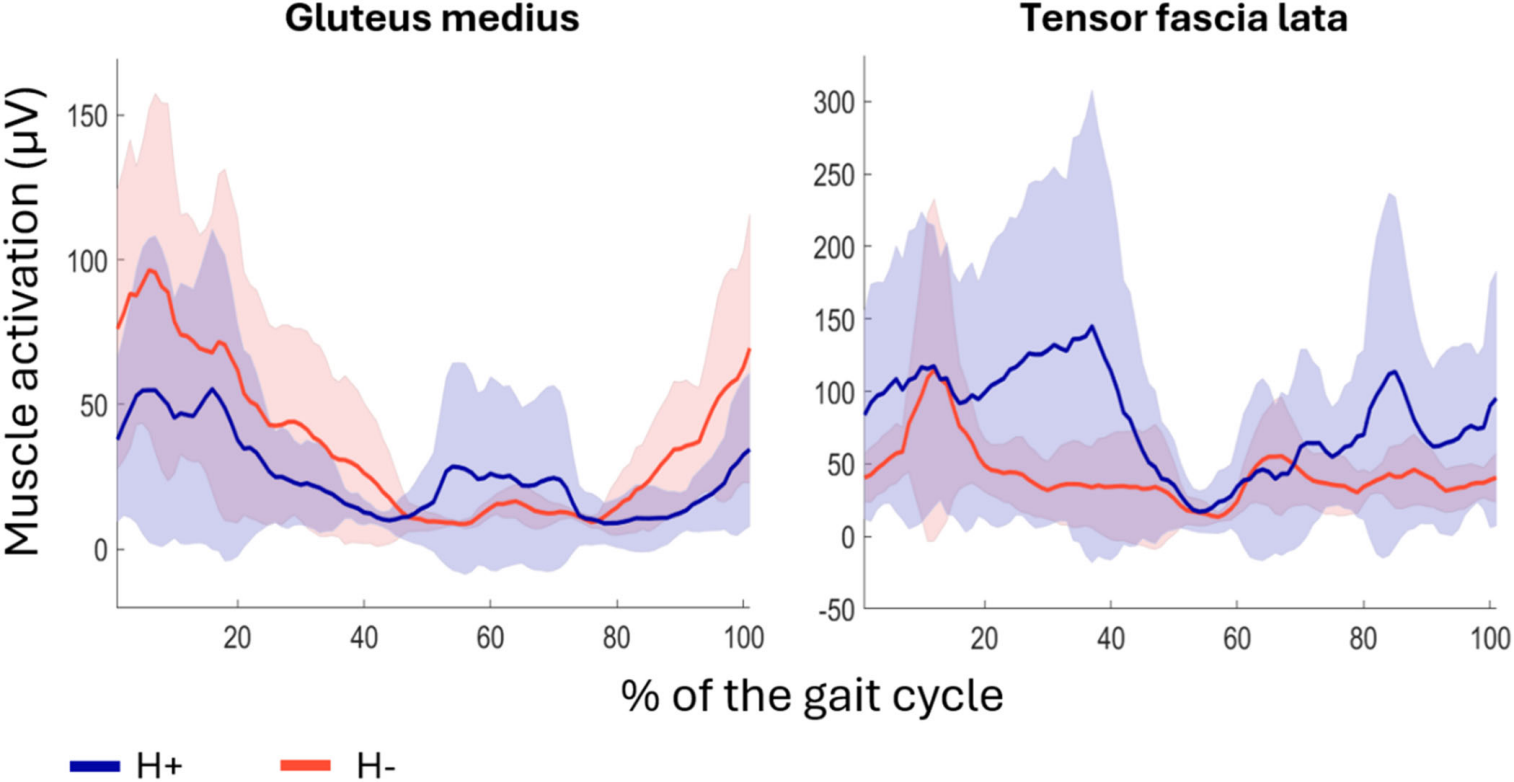
EMG activation of the gluteus medius and tensor fascia lata over the gait cycle. Solid lines represent the mean muscle activation of the Gmed and TFL hypertrophy (H+) and no hypertrophy (H−) groups. The lighter coloured areas depict standard deviation. EMG, electromyography; Gmed, gluteus medius; TFL, tensor fascia latae.

GMed activation tended to be slightly greater during the stance phase for the H− group, while the TFL was most activated in the H+ group in the same period (Figure 6).

## DISCUSSION

Reconstruction of the proximal femur after bone tumour resection can lead to gait impairment, mostly due to loss of abductor integrity, failure of the abductors to heal to the prosthetic device or incomplete adaptive motor compensation. The objective of this study was to evaluate whether the tenodesis of abductor muscles following proximal femoral resection can lead to TFL hypertrophy and whether this hypertrophy would result in better functional outcomes for these patients. The soft tissue reconstruction technique was performed on 16 patients, 50% of whom experienced radiographic TFL hypertrophy. The healing of the tenodesis was not assessed through imaging, as metal artefacts in CT and MRI significantly limit the ability to visualize the tenodesis site adequately. In several reviewed cases, including one patient from this study, extensive scarring has been observed between the TFL and gluteus medius muscle layers. Furthermore, the two tendons are found to be inseparable and firmly attached to the prosthesis.

Patients with TFL hypertrophy demonstrated higher patient‐reported outcome scores across all measures, matching or exceeding values reported in the literature, despite our cohort's older age. Four of eleven patients (36%) were excluded from gait analysis because they required assistive devices, a rate that aligns closely with the 30.4% ambulation‐assistance reported for soft‐tissue repairs in a recent meta‐analysis [30]. Rompen et al. [33] adapted the ambulation score, originally developed for amputees, for lower‐extremity endoprosthetic reconstruction. They sutured the gluteal muscles to the tensor fascia lata and vastus lateralis remnants in proximal and total femoral procedures. They observed mean A1 and A2 scores of 47% and 25%, respectively, compared with 69% and 68% in our cohort; their lower results likely reflect the inclusion of longer distal femoral resections. Other studies prefer using the TESS to assess subjective function. In a study of 100 patients, Chandrasekar et al. reported a mean TESS of 61% while Ogilvie et al. described a TESS of 76.2% in patients undergoing either soft‐tissue or greater trochanter reconstructions [7, 31]. In this study, the patients with TFL hypertrophy had a mean TESS of 77%.

In the present study, the H+ group had an MSTS score of 84%. By comparison, Thambapillary et al. reported a mean MSTS of 68.7% in 130 patients treated with cemented proximal femoral endoprostheses using various abductor repair techniques [36]. Giurea et al. found a score of 64% after gluteus medius reinsertion in six patients, and Ogilvie et al. observed a score of 67.7% [16, 31]. Benedetti et al. compared MEP and the allograft‐prosthesis composite. In APC, both gluteus medius and iliopsoas were reattached to an allograft tendon, similar to our method. In MEP, only the gluteus medius was reattached via a trochanter device [3]. They observed MSTS scores of 87% (MEP) and 90% (APC), though their cohort was 20–30 years younger than ours. Given that MEPs are more readily available than APCs, a direct comparison of abductor function after APC versus endoprosthesis reconstruction using the same tenodesis technique would be highly informative. The limited data to date suggest our surgical method may perform on par with APC reconstruction.

Zhang et al. wrapped the prosthesis in synthetic ligament, sutured the muscle stump anatomically onto its surface, and used the ligament as the attachment carrier [37]. They reported an MSTS of 89.3% and a Harris Hip Score of 90%, with all patients regaining normal gait. These superior scores likely result, in part, from cohort differences, namely a younger mean age (40 years vs. >60 years) and shorter resection lengths (9 cm vs. 14 cm). Building on this principle, adding a mesh around the prosthesis could promote tendons' ability to heal around the prosthesis and form a robust scar‐tissue sleeve enveloping the gluteus medius, tensor fascia lata and vastus lateralis. Such a sleeve could enhance abductor function by creating a stronger soft‐tissue interface than metal alone. Future research could explore the combined use of tenodesis and mesh augmentation to assess their potential for improving abductor strength and overall hip function.

Gait pattern analysis highlighted both similarities and divergences between the H+ and H− groups. In the sagittal plane, joint angles of the hip, knee and ankle were similar; this was expected since the surgical technique did not address the quadriceps or the iliopsoas. Benedetti et al. and Rompen et al. described maintenance of a greater hip extension at toe‐off during walking and a stiff knee gait in patients with a femoral prosthesis after tumour resection compared to controls or the non‐operated leg, respectively [3, 33]. These characteristics of gait were more apparent in the H− patients, who exhibited on average less hip extension, knee flexion and ankle plantarflexion than H+ patients. H+ patients were comparable to healthy individuals in this regard [15]. Despite this, additional findings from the gait analysis suggest that the group with TFL hypertrophy may have an overall weaker abductor mechanism compared to the group without TFL hypertrophy. One key indicator is the notable difference in hip adduction and pelvic obliquity during single‐limb support. Patients in the H+ group tended to have a greater pelvic drop, for a greater percentage of the gait cycle. This was an objective measurement of a more pronounced Trendelenburg gait. One reason for this outcome may be that having a smaller abductor mechanism is what elicited TFL hypertrophy, as a means to compensate. This is especially apparent when observing the interaction effect in muscle CSA of the GMax, and the GMed following a similar trajectory: it appears that for both muscles on the surgical side, the H− group had more to lose, did lose more, yet still ended up with greater CSA. In short, patients who experienced hypertrophy of the TFL might have been more incapacitated to begin with; the tenodesis and resulting hypertrophy of the TFL might have helped compensate to a point, potentially explaining the greater reliance on TFL activation rather than GMed during the stance phase. This hypothesis cannot be confirmed by the present study. Further research is needed to clarify which patients may be better candidates for developing hypertrophy and whether it improves their condition from baseline.

While this study provides initial insights into a newer surgical technique that may maximise the usability of the TFL in an abductor role, it remains uncertain what factors influence the development of hypertrophy in some patients. Hypertrophy would occur due to greater stimulus to the reinserted muscle, be it from a different recruitment pattern or a change in muscle fibre orientation from the tenodesis effect. Training and physical therapy are likely not the only factors responsible for the TFL hypertrophy since all patients participated in specialized rehabilitation program and they all developed similar amount of muscle FI on the affected side. As both groups had less FI in the TFL on the operated side, it can be surmised that both groups benefited from this program, whether it resulted in hypertrophy of the muscle or not. It was thought that the tenodesis effect could affect the GMed activity by also providing another anchor point to the abductor mechanism. The fascia latae would then act as another fixed point that helps stabilize the pelvis. The result of the present study tends to contradict this hypothesis, as there was no benefit to pelvis stabilization for patients with TFL hypertrophy.

### Limitations

This study is limited by its small cohort size, a constraint largely driven by the oncological context. Many patients did not survive long enough to become eligible for inclusion. Some data, specifically tumour size and soft tissue involvement, were inconsistently reported in patients' medical files. To address this, we used resection length and the documented occurrences of gluteus medius and quadriceps resections as proxy measures, confirming that the study groups were equivalent based on these metrics. However, given the rarity of this patient population, a degree of heterogeneity is unavoidable, with factors such as patient age, resection length, comorbidities, and the extent of soft tissue resection influencing functional outcomes. These variables should be carefully considered when interpreting results, particularly in relation to gait mechanics and compensatory muscle activation. Furthermore, the study's retrospective design precluded a preoperative assessment of function, which would have been valuable for meaningful group comparisons.

The absence of a control group is another limitation of this study. To address this, comparisons were made between each patient's affected side and their non‐operated side, allowing each patient to serve as their own control. Finally, the presence of prosthesis artefacts on CT made the exact contouring of the muscle on some cross‐sections difficult. However, the intraclass correlation was 0.84–0.97, meaning that it did not affect the radiological measures substantially.

## CONCLUSION

The reorientation of the TFL by a tenodesis with the abductor mechanism to the MEP led to TFL hypertrophy in half of the patients with proximal femoral reconstruction. This was not associated with significant objective functional benefit, although subjectively, patients with hypertrophy reported satisfactory outcomes. Differences in gait pattern did not favour the group with hypertrophy, who exhibited greater pelvic instability. In conclusion, TFL tenodesis appears to be a viable surgical technique that may provide functional benefits regardless of the presence of TFL hypertrophy. A longitudinal comparative study is necessary to determine whether this approach offers advantages over more widely adopted surgical techniques.

## AUTHOR CONTRIBUTIONS

**Ariane Lavoie‐Hudon:** Data curation; formal analysis; visualization; writing—original draft. **Jean‐Philippe Cloutier:** Investigation; data curation. **Norbert Dion:** Conceptualization; writing—review and editing. **Simon Laurendeau:** Investigation. **Philippe Corbeil:** Supervision; writing—review and editing. **Annie Arteau:** Conceptualization; supervision; writing—review and editing.

## CONFLICT OF INTEREST STATEMENT

The authors declare no conflicts of interest.

## ETHICS STATEMENT

All procedures in this project were performed in compliance with relevant laws and institutional guidelines and have been approved by the institutional ethics committee. Research ethics board of the CHU de Québec–Université Laval; Approval number: 2016‐2928, obtained on 5 November 2016. All participants provided their written informed consent prior to participating, and their rights to privacy have been observed.

## Supporting information

Supplementary Material Anonymized.

## Data Availability

The data that support the findings of this study are available from the corresponding author upon reasonable request.
