# Supplementary material for: Does tenodesis of tensor fascia latae with hip abductors after proximal femoral resection and modular endoprosthetic reconstruction lead to functional improvements?
Source: J Exp Orthop. 2025 Dec 7;12(4):e70534. doi: 10.1002/jeo2.70534 (PMC12682228; doi:10.1002/jeo2.70534)
Supplement: Supplementary file 1 — Supplementary Material Anonymized. [file JEO2-12-e70534-s001.pdf]

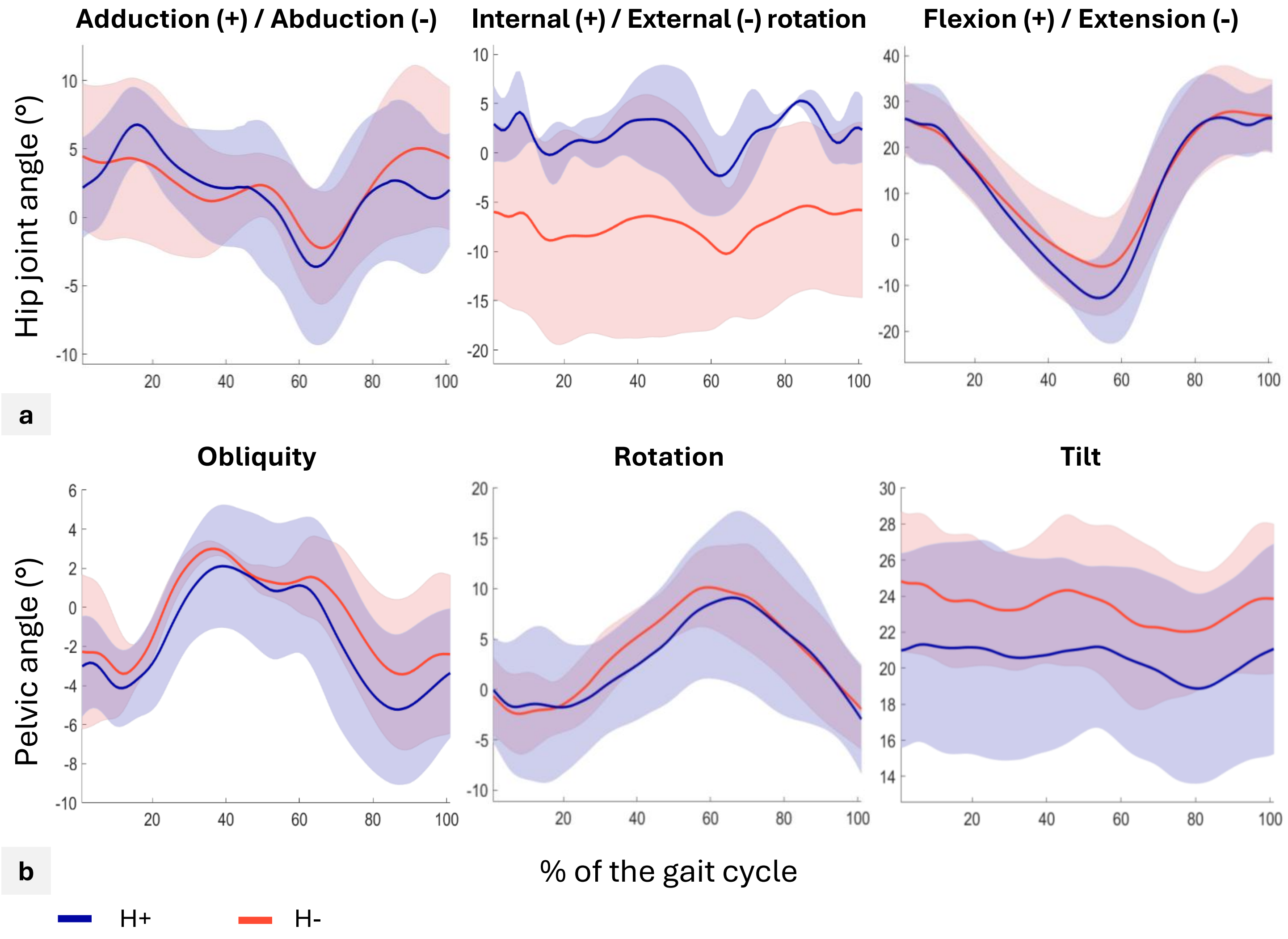

Supplementary Fig. 1a-b Kinematics of the healthy hip and the pelvis over the gait cycle. Solid lines represent the mean joint angles of the operated hip (a) and the mean pelvic angle (b), for the tensor fascia latae hypertrophy (H+) and no hypertrophy (H-) groups. The lighter colored areas depict standard deviation.

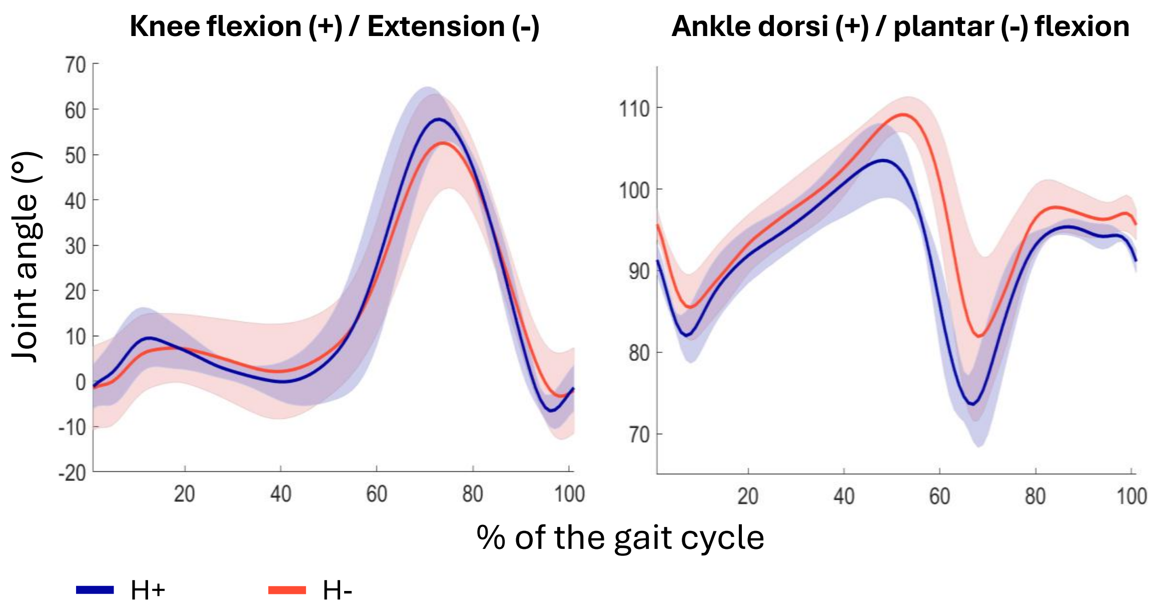

Supplementary Fig. 2 Kinematics of the healthy knee and ankle over the gait cycle. Solid lines represent the mean joint angles of the knee and ankle for the tensor fascia latae hypertrophy (H+) and no hypertrophy (H-) groups in the sagittal plane. The lighter colored areas depict standard deviation.

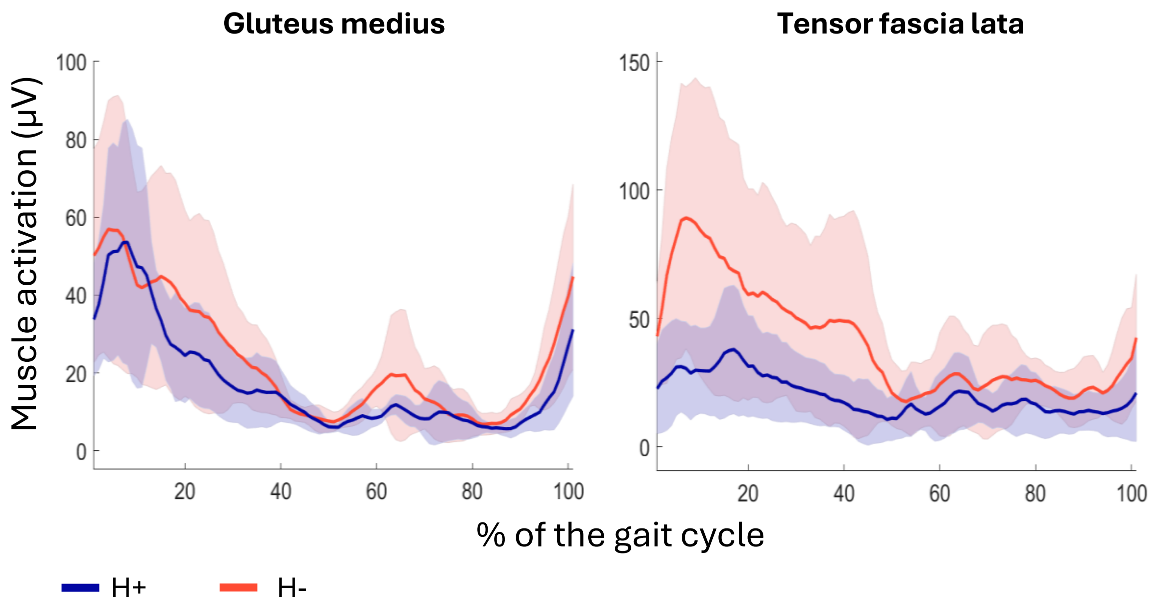

Supplementary Fig. 3 EMG activation of the gluteus medius (GMed) and tensor fascia latae (TFL) over the gait cycle. Solid lines represent the mean muscle activation of the GMed and TFL for the hypertrophy (H+) and no hypertrophy (H-) groups. The lighter colored areas depict standard deviation.
